# Implications of Covid-19 Pandemic on School-age children with Autism Spectrum Disorders

**DOI:** 10.1192/j.eurpsy.2022.756

**Published:** 2022-09-01

**Authors:** I. Farmakopoulou, C. Efstratiou, A. Dokos, E. Lepsinioti, M. Theodoratou

**Affiliations:** 1 University of Patras, Humanistic Sciences, Patras, Greece; 2 University of Patras, Humanistic Sciences, Rio, Patras, Greece; 3 Hellenic Open University, Human Sciences, Patras, Greece; 4 Hellenic Open University, Human Sciences, PAtras, Greece; 5 Neapolis University of Pafos, Health Sciences, Pafos, Cyprus

**Keywords:** covid-19 pandemic, Family, Autism spectrum disorder (ASD), school- aged children

## Abstract

**Introduction:**

The Covid-19 pandemic has caused multilevel changes worldwide. Everyday life of all people has been altered drastically. Children with ASD seem to face difficulties due to their heightened sensitivity to unpredictable and complex changes in their lifestyle. Our presentation aims to reveal the effects of Covid-19 on school-age children with Autism Spectrum Disorders.

**Objectives:**

The main thematic areas of this research, concerning the social workers’ questionnaire, focused on the routine, psycho-emotional field, school performance, sociability and school environment of children with ASD.

**Methods:**

Between June 22nd and August 16th, 2021, social workers (n=38) and parents (n=25) administrated a questionnaire -through google form platform- which investigated issues around routine, psycho-emotional field, school performance, sociability, school, and family. Descriptive statistics were used for statistical analysis of the data.

**Results:**

The routine and psycho-emotional conditions of children with ASD were found to have a negative shift. Moreover, the degree of change on school performance was moderate, while sociability change was minimal. The significance of the school’s contribution and the impact of the Covid-19 pandemic on the family functionality was highly observed. Another interesting finding was that children with ASD did not display discomfort for the mandatory social limitations or fear for the virus transmission.

**Conclusions:**

Lastly, it is important to implement appropriate practices for the protection of children with ASD, through the mobilization of the responsible parties and thus, social policy transformations are vital for this vulnerable population.

**Disclosure:**

No significant relationships.

